# The role of plumage and heat dissipation areas in thermoregulation in doves

**DOI:** 10.1242/jeb.248200

**Published:** 2025-02-20

**Authors:** Kristen E. Crandell, Donald R. Powers, Bret W. Tobalske

**Affiliations:** ^1^ School of Environmental and Natural Sciences, Bangor University, Gwynedd LL57 2UW, UK; ^2^ Biology Department, George Fox University, Newberg, OR 97132, USA; ^3^ Field Research Station at Fort Missoula, Division of Biological Sciences, University of Montana, Missoula, MT 59812, USA

**Keywords:** Hyperthermia, Thermal gradient, Flight, Bird, Feathers, *Streptopelia risoria*, Thermal window

## Abstract

Avian plumage contributes to the regulation of body temperature. In most climates, avian heat dissipation occurs passively via radiation, conduction and convection owing to the thermal gradient between the environment and the animal. The muscles that power flight also produce significant heat that must be dissipated. How plumage and areas with sparse or no feathers (termed ‘heat dissipation areas’, HDAs) interact with these mechanisms is unclear. We examined the role of plumage as an insulator, or dissipator, of heat in ringed turtle-doves (*Streptopelia risoria*) under four thermal regimes: resting, post-flight, heating via radiative lamps, and cooling via wind. We measured internal body temperature and skin-level temperature (under the plumage) using thermal PIT tags alongside surface temperature using a thermal imaging camera. Flight increased internal temperature by 0.6°C compared with resting, but the other treatments did not have significant effects. The skin-level temperature during wind exposure was 1.6°C cooler than in other conditions. HDAs changed in surface area above 35°C but not maximum temperature among treatments. Post-flight and during radiant heating, birds increased HDA surface area – most notably at the wing. During simulated wind produced using a fan, the HDAs of the beak and wing were eliminated, and areas of other HDAs were reduced. Our results demonstrate that birds modulate active HDAs to maintain consistent core body temperatures under induced temperature challenges. They also promote caution for extrapolating from thermal images of surface temperature to infer core temperature in birds.

## Teaser

**Editors' choice:** Modulations in heat dissipation areas contribute to thermoregulation in doves exposed to external thermal regimes and after flight exercise; caution is warranted when inferring core temperature from skin-level or external measures.

## INTRODUCTION

Avian plumage serves multiple roles, including social signalling, camouflage, flight and regulating body temperatures (Terrill and Shultz, 2023). Thermoregulation combined with maintaining basal metabolic rate can take up to 60% of a bird's daily energetic expenditure (Walsberg, 1983). Recent studies have revealed the importance of the integument and plumage for avian thermoregulation. Both plumage (Wolf and Walsberg, 2000) and areas with sparse or no feathers, termed heat dissipation areas (HDAs; McCafferty et al., 2011), play an integral role in this process.

Hyperthermia, wherein body temperatures rise above physiologically acceptable limits, can quickly prove lethal and is a growing concern in our warming world. Recent studies have revealed that birds trade off breeding success, and even self-maintenance, to minimize or avoid exceeding their acceptable thermal limits. For example, both the southern pied babbler (du Plessis et al., 2012) and the little bustard (Silva et al., 2015) reduce activity levels, and spend more time actively dissipating heat, as ambient temperatures rise to and exceed body temperature. Conversely, relaxing insulative barriers to heat dissipation by experimentally trimming feathers may increase fitness. Tree swallows (Tapper et al., 2020) and blue tits (Nord and Nilsson, 2019) that had contour feathers over their abdomen trimmed, and thus spent less time offloading heat, had heavier chicks. The swallows additionally had higher fledging rates and more frequent feeding events at the nest. These results lend support to a ‘heat dissipation limitation’ hypothesis (Speakman and Król, 2010).

The source of excess heat can be from either the animal or the environment. Internally, body heat is produced as a by-product of mechanical work and biochemical reactions. Because flapping flight is the most costly form of vertebrate locomotion in terms of power requirements (Butler, 1991; Alexander, 2002), heat dissipation is crucial. Flight muscle efficiency (mechanical work output/metabolic energy input) is between 10 and 15%, and the majority of the remaining 85–90% of metabolic work is dissipated as heat (Welch et al., 2007; Wells, 1993). Birds dissipate heat via convection during flight (Powers et al., 2015); however, the limited available evidence suggests birds must continue to dissipate a heat load built up during flight after they land (Lewden et al., 2023; Schraft et al., 2019).

Environmental heat input can rapidly rise 10-fold or more above the average basal metabolic rate of a bird owing to solar radiation (Wolf and Walsberg, 2000). Plumage colour and structure can deflect, or absorb, visible wavelengths (Walsberg et al., 1978; Wolf and Walsberg, 2000). If external temperatures are above the organism's temperature, heat loss is behaviourally maintained (Pattinson et al., 2020), often by seeking cooler areas including shade (Bourne and Soravia, 2023), or by reducing maintenance or breeding behaviours (Bourne et al., 2023; Silva et al., 2015). Active heat dissipation behaviours include panting to increase evaporative water loss (Gerson et al., 2014). The rate of radiative heat loss at an HDA is also increased (Powers et al., 2015; Ward et al., 1999) via increasing blood flow in the beak (Tattersall et al., 2009), exposing the legs (Martineau and Larochelle, 1988), and wing extension and elevation (‘drooping’; Arad et al., 1989). Relying on environmental or behaviourally induced convection and conduction mechanisms, for example during flight, contributes to active heat dissipation as well (Lewden et al., 2023).

Maintaining core body temperatures in cold conditions can be just as challenging. Plumage is well known to play a key role in slowing the loss of heat produced by the body to the environment (Lucas and Stettenheim, 1972). Heat retention is facilitated by trapping air between the skin and plumage, creating an insulating layer (Dove and Agreda, 2007). Plumage morphology across species is associated with local environment – species in colder climates have longer, less dense barbs in the body (contour) feathers, allowing for increased volume of trapped air (Pap et al., 2020). In addition, decreasing blood flow to areas of integument not covered by feathers, most notably the beak, may help maintain body temperature. Before or concurrent with reducing local blood flow, birds often behaviourally adjust by covering the exposed regions by tucking their beaks under plumage or changing posture to cover their legs (Tattersall et al., 2017).

Feathers can manage both retaining and offloading heat in part because the feather layer is behaviourally controlled. A bird can increase the insulating layer volume, and thus insulative properties, by ptiloerection. A bird can increase skin surface area exposure by active moving feathers during preening (McFarland and Baher, 1968; McFarland and Budgell, 1970). Alongside modifying feather position and function behaviourally, birds also use areas of exposed skin, the beak and legs to offload additional heat. These patches are called HDAs (McCafferty et al., 2011). These areas are controllable and adjustable (Tattersall et al., 2009), allowing birds to shunt heat via increased blood flow to the surface, or retain heat by vasoconstriction.

Consequently, birds offer a unique opportunity to examine the maintenance of core body heat via behavioural and physiological modifications of both the thermally variable function of the feathers and the modulation of HDAs. In this study, we experimentally manipulated thermal regimes while measuring core body temperature and skin-level temperature using thermal PIT tags and surface temperature using infrared thermography. Birds were tested under a radiative heat treatment, a wind-induced cooling treatment, a post-flight regime and resting conditions. We tested for variation in thermal gradients owing to feather insulation and for modulation of thermoregulation via variation in HDAs. In all treatments, core body temperatures were predicted to be highest, with a scaled decrease in temperature between the skin and plumage relative to external measures. In heating treatments (radiative heat and post-flight), we predicted increased surface areas above a 35°C threshold across all HDAs and increased average and maximum temperatures observed in these regions relative to resting. We likewise predicted a notable decrease or absence of surface areas above a 35°C threshold during the cooled treatment, with lower average and maximum temperature values.

## MATERIALS AND METHODS

### Birds and preparation

Nine captive Eurasian collared doves [*Streptopelia risoria* (Linnaeus 1758), body mass=162±13 g, sex and age unknown] were used in this study in accordance with the University of Montana IACUC approved AUP 031-22BTDBS-061322. Birds were housed in an indoor aviary (average temperature 23°C, 12 h:12 h light:dark cycle), and fed and provided water *ad libitum*. Animals were identified individually using coloured leg rings. Birds were individually implanted with a thermal PIT tags (Biotherm 13, 13×2.12 mm FDX-B 134.2 kHz; Biomark, Boise, ID, USA) in the abdominal coelom immediately caudal to the apex of the keel of the sternum, approximately 1 cm deep using a Biomark MK10 PIT Tag Implanter and N125 needle. Prior to implanting the overlying skin was anesthetized using lidocaine, and the skin opening was closed using cyanoacrylate glue. Birds were allowed to recover and rest for at least 1 day following insertion before experimental procedures. Immediately before the experiments, we used cyanoacrylate glue to attach a second Biotherm 13 PIT tag to the skin immediately adjacent to the left sternal feather tract (pterylae) at the midpoint of the sternum.

### Experimental protocol

All trials were run in an environmentally controlled room (thermal chamber) with inner dimensions of 2.4×2.4×2.4 m at the Field Research Station at Fort Missoula (University of Montana, Missoula, MO, USA), with ambient temperatures maintained at 23±2°C. We obtained PIT tag and thermal imagery with the doves perched on a wooden dowel mounted on a tripod. We recorded from the thermal PIT tags using a Biomark HPR Plus Handheld PIT Tag Reader with a Racket Antenna mounted below the perch. The reader sampled at 8 Hz and data were transmitted via a USB cable to a computer and recorded using Biomark Bioterm software. To monitor ambient temperature, we used K-type thermocouple wire implanted into a plastic ping-pong ball that was coated first using copper paint and then using flat grey paint. The thermocouple wire was connected to an Omega HH520 data logger (Omega Engineering, Inc., Norwalk, CT, USA) measuring at 8 Hz, connected to a computer via USB, and data were recorded using Omega Testlink software. We used a forward looking infrared (FLIR) T650SC video camera sampling at 10 Hz (680×420 resolution; Teledyne FLIR, LLC, Wilsonville, OR, USA) to measure surface temperature of the doves. Immediately before the experiments, we had the camera calibrated by Teledyne FLIR to an accuracy of ±1°C or 1% of reading for an object temperature range of 5°C to 120°C and ambient temperature range of 10°C to 35°C. The noise equivalent temperature difference (NETD) sensitivity of the camera is rated by Teledyne FLIR to be <0.02°C. We placed the camera to provide a lateral view of the birds at 1.5 m distance from the perch. Camera images were calibrated to metric scale daily using a metal ruler held parallel to the lens at the perch position.

### Thermal treatments

Birds were brought into the thermal chamber one at a time and allowed to habituate on the perch for 10 min prior to any treatments occurring. Experimental protocols only occurred if the animals appeared calm. We randomized treatment order to avoid potential bias in sequence of thermal conditions.

Resting measurements were taken following 10 min of quiet, unperturbed perching. Radiant heating treatments were accomplished using one Westinghouse 250 W R40 Reflector Heat Lamp mounted in an ACE 300 W Clamp Light housing directly illuminating the front of the bird from a distance of 0.5 m. Cooling treatments were accomplished using a custom-built fan that consisted of four individual, fixed-RPM 12 V DC fans each 15 cm in diameter so that total fan outlet measured 30×30 cm. Outlet flow was rendered laminar using a sheet of aluminium honeycomb 2.5 cm thick with hexagonal openings 3.2 mm in diameter. The fan was placed 0.5 m in front of the bird, and average wind speed at this distance was 1.7 m s^−1^. Both radiant heating and fan treatments ran for 10 min, with the bird at rest on the perch. FLIR recordings and PIT tag readings were taken immediately once the treatments ceased.

The post-flight treatment was accomplished by manually transferring the dove on a second perch to the opposite side of the thermal chamber (approximately 1.5 m away from the resting perch), and encouraging flight to the perch. The bird flew 10 times in quick succession, with a total flight time of less than 3 min. Doves had been trained to independently take off from a hand-held perch on the other side of the chamber and land on the recording perch, from which they were collected immediately to fly again. Flight training prior to the experiment was used to minimize the potential for stress, and so minimize any effects of stress on core and surface temperature during this treatment. Thermal and PIT readings were taken immediately after the 10th flight, with the bird at rest on the perch.

### Data extraction

#### FLIR

**Fig. 1.**
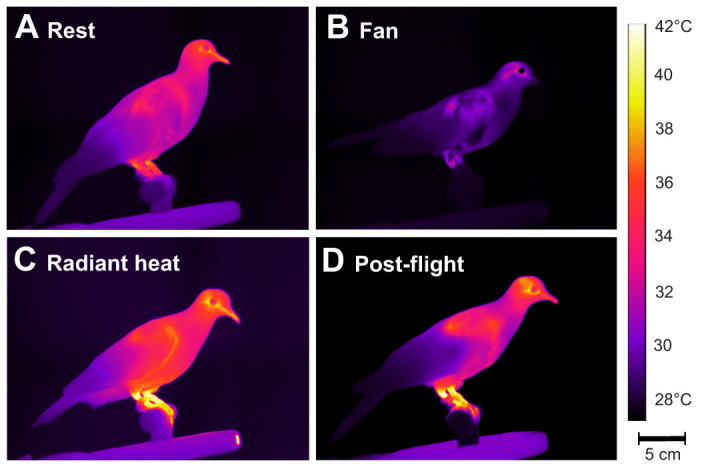
**Illustrative FLIR images for an individual Eurasian collared dove (*Streptopelia decaocto*) taken immediately following treatment.** (A) Resting, (B) fan, (C) radiant heat and (D) post-flight. Differences in heat dissipation area (HAD) surface area and temperature (°C) are apparent among treatments.

FLIR data were initially analysed using FLIR ExaminIR software. We viewed the first minute of post-treatment video to sample a single frame where the animal held motionless on the perch and oriented posture with its long axis parallel to the focal plane of the camera. Tabh et al. (2021) found that FLIR measures were susceptible to orientation, so our selection was careful to maximize the lateral projected area of the perched bird. Suitable frames of video were exported as text images and subsequently analyzed using ImageJ (Fiji v2.15.0, National Institutes of Health, Bethesda, MD, USA) as in Powers et al. (2017). Within ImageJ, for measures of surface area of the HAD, we chose a surface temperature threshold ≥35°C to delimit HDAs, as this was the minimum temperature at which the region boundaries were clearly defined. This threshold allowed us to compare values for surface area of HDA across treatments. Surface area and temperature mean and maximum values were taken at the same time. Temperature values were extracted using 35°C thresholding as a baseline, but in instances of cooler treatments (<35°C), thresholding was identified as visually distinct areas as in Powers et al. (2015). The HDAs used in our analysis were: face (including the eye and beak regions), wing (including the surface under the wing exposed if the wing is slightly elevated and extended, i.e. ‘droops’) and feet (see Fig. 1). There is a likelihood that surface area and mean temperature correlate, given that one is defined by a threshold of the other. We tested for correlation within each of the HDAs using a linear regression, and found that there existed a correlation between the mean temperature and surface area for the foot HDA (*r*^2^=0.19, *P*<0.01) and wing HDA (*r*^2^=0.58, *P*<0.001), but not the face HDA (*r*^2^=0.01, *P*=0.46). Patterns for the foot and wing are expected given that offloading heat is likely a combination of increasing overall surface area as well as temperatures at the HDAs (Powers et al., 2015).

Two additional measurements were extracted from the thermal images. The first was a measure of external temperatures of the entire lateral view of the whole animal, identified via thresholding between the animal and its environment. This measure excluded the tail (the plumage beyond the body of the animal) as the distal ends of it were often not distinguishable from ambient temperature. The second measurement taken was an average temperature for 1 cm^2^ selected along the ventral surface at the approximate location of the skin-level pit tag, to ascertain the gradient from surface-level temperatures above the skin-level tag.

#### PIT tags

A single data point within 30 s of the end of the experimental treatment was extracted after each treatment for both internal and external PIT tag readings. In the event of temperature fluctuation within the 30 s interval, maximum values were taken for treatments wherein maximum temperature was most relevant (resting, radiant heating and post-flight), whereas minimum values were taken for fan treatment.

### Analysis

Data were analysed in R (https://www.r-project.org/). To examine the functional gradient from core to skin-level to external surface temperature, we ran an ANOVA among the three measurements with individual bird and thermal treatment as factors.

To explore the differences between thermal treatments, a repeated-measures ANCOVA was run between treatments, with individual bird as a factor. Both ‘treatment’ and ‘HDA’ were treated as fixed factors, and the potential interaction between HDA and individual bird was an error term.

We additionally used the R package Thermimage (https://CRAN.R-project.org/package=Thermimage) to model and estimate the rate of heat loss (W m^−2^) for each of the HDAs, as described in Tattersall et al. (2018). For these estimates, we assumed a negligible conductive heat loss via the foot–perch contact, and negligible evaporative losses. A surface reflectance of 0.1 was used, based on estimates for finches (Tattersall et al., 2018). Animal tissue emissivity of 0.96 was assumed (Tattersall et al., 2018). The HDAs were modelled with a simplifying flat-plate geometry. Estimated total rate of heat loss summed area-specific convective heat transfer and radiative heat flux.

To determine whether infrared image data are a reliable representation of internal core body temperatures, we ran a step-wise AIC analysis on the linear model of HDA temperature and internal temperatures using the R library MASS (Venables and Ripley, 2002). To better reflect measurements taken on wild populations, wherein attaining accurate internal estimates based solely on FLIR data is most likely to occur, individual bird ID was not used as a factor – the presumption being that wild individuals are more challenging to identify to individual in thermal imaging data.

To further examine the relationship between a representative HDA and core body temperature, we used maximum temperatures for the face HAD, aligning with recent lab and field-based studies that have used this area as representative of the animal (Bakken et al., 2005; Edgar et al., 2011; Tabh et al., 2021). We ran a linear mixed effects model using the R package lme4 (Bates et al., 2015), with eye HDA maximum temperature and thermal treatment as fixed effects. The interaction between them was additionally modelled, and individual bird was treated as a random intercept. Model selection and Akaike's information criterion corrected for small sample size (AICc) ranking was accomplished using the MuMln package (https://cran.r-project.org/web/packages/MuMIn/index.html), to reduce human error in model construction. Models were ranked using AICc, with model weight over 2% reported (e.g. delta AICc<2). Marginal and conditional *r*^2^ values were calculated for the highest-ranking models (Nakagawa and Schielzeth, 2013).

## RESULTS

### Gradient between core, skin-level and external temperatures

**Fig. 2.**
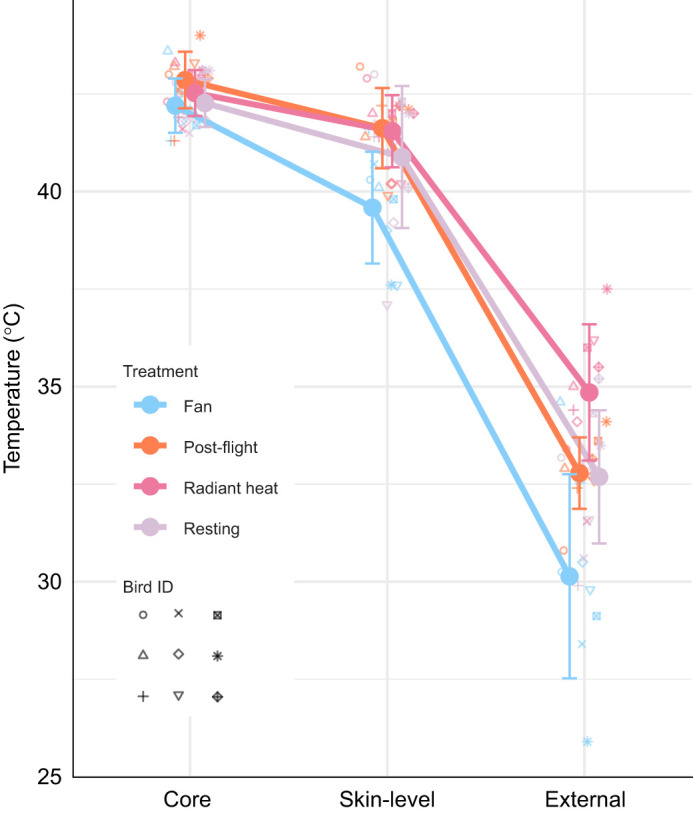
**Temperature (°C) gradient from PIT tag implanted at the core of the bird, PIT tag above the skin but below the plumage layer, and external temperatures measured from the IR camera.** The external measurements were taken as a 1 cm^2^ average temperature along the ventral surface, at the approximate location of the skin-level PIT tag. Colours represent the four thermal treatments. Each large point represents the mean, with error bars representing one standard deviation for that measured location. Means are connected by lines within a treatment. Individual bird ID (*n*=9) data are represented by nine symbols. Points are slightly jittered on the *x*-axis for ease of visibility – the jitter is standardized for each location such that slopes of the lines remain directly comparable. Each measured location was significantly different from the others (ANOVA, *P*<0.001, see Results for details).

A statistically significant gradient existed between the core, skin-level (i.e. sub-plumage) and external temperature (Fig. 2). Measurements of core body temperature were significantly warmer than measurements of skin-level temperature (ANOVA with individual bird and treatment as factors: *F*_1,60_=35.05, *P*<0.001). Skin-level temperatures were significantly warmer than external IR-measured temperatures above the plumage (ANOVA with individual bird and treatment as factors: *F*_1,60_=302.9, *P*<0.001).

### Both internal body temperature and skin-level temperatures respond to treatments

Internal body temperature varied with treatment (ANOVA with individual as a factor: *P*=0.009; Fig. 2). Post-flight birds had measurably higher internal temperatures (mean 42.9±0.7°C) when compared with the fan treatment (mean 42.1±0.7°C, Tukey HSD *P*<0.01) and resting (mean 42.3±0.6°C, *P*=0.04). Post-flight and radiant heating (mean=42.5±0.6°C) treatment birds were not statistically different (*P*=0.40).

Skin-level temperature varied with treatment (ANOVA with individual bird as a factor: *P*<0.001). Skin-level temperature was significantly lower in the fan treatment (mean 39.3±1.6°C, Tukey HSD *P*<0.01). Mean values for post-flight, radiant heating and resting were not significantly different from each other (*P*>0.05, mean post-flight=41.6±1.0°C, radiant heating=41.5±0.9°C, resting=40.9±1.8°C).

### HDAs change temperature and surface area in different treatments

**Fig. 3.**
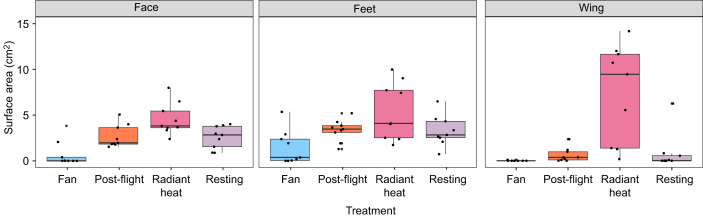
**Surface area (cm^2^) of the three main HDAs (*n*=9 doves), measured as any active surface above 35°C, from a lateral view.** Graph is colour coded by treatment. Compared with resting, surface areas increased post-flight and with a heat lamp (radiant heat), and decreased or were not present in the fan treatment (*P*<0.001, see Results for details).

**Fig. 4.**
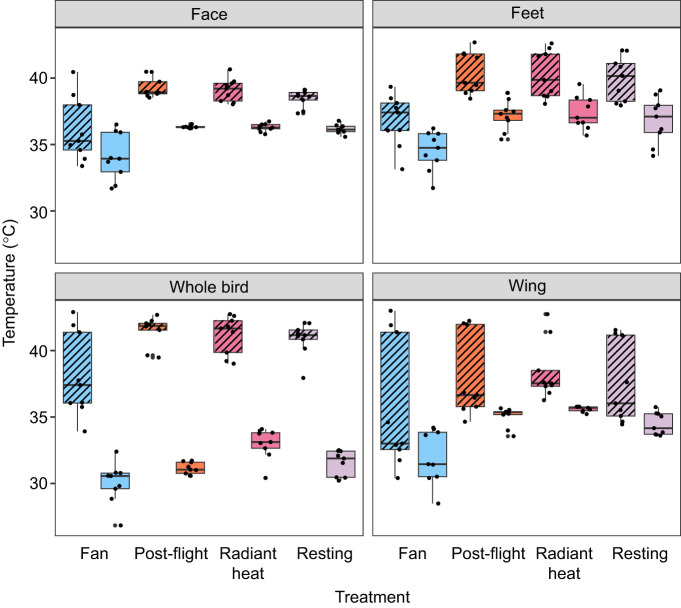
**Average (solid colour) and maximum (hashed colour) temperatures (°C) observed for each HDA (face, feet and wing) measured for nine doves.** The ‘whole bird’ measurement is average temperature across the entire lateral view of the animal, excluding the tail, and is used as a representation of overall temperature differences that may not be encompassed within the HDAs. Graph is colour coded by treatment. For statistical results, see Results.

Treatment had a significant effect on surface area of active HDAs (repeated-measures ANCOVA: *P*<0.001; Fig. 3) and mean external temperature of the HDAs (*P*<0.001; Fig. 4). Birds appeared to modulate both the temperature of the HDAs and surface area (Fig. 1).

For the fan treatment, both temperature and surface area of the active HDAs were modulated. Surface temperature of the overall bird was cooler than resting conditions (Fig. 4). HDA temperatures were lower in fan treatments in comparison with resting levels at the eye (*P*<0.001), feet (*P*=0.003) and wing (*P*=0.04) (Fig. 4). Surface area of the HDA was significantly smaller at the eye (*P*=0.04), but not at the feet (*P*=0.26) or wing (*P*=0.89) in comparison with resting. Overall, lower external body temperatures appeared to be due to changes in HDA mean temperatures and overall surface area of the HDAs in comparison with resting conditions.

For the radiant heating treatment, external bird temperatures were overall warmer in comparison with resting (*P*=0.03). However, active HDA mean temperatures in the eye, feet and wing did not differ significantly from resting values (*P*>0.05 for all). Surface area values of the active HDAs did differ, and were larger than resting values at the eye (*P*=0.03) and wing (*P*<0.0001), but not the feet (*P*=0.12). Thus, the external temperature difference appeared to not be due to changes in active HDA mean temperatures, but instead to overall surface area of the HDAs in comparison with resting conditions.

For post-flight, bird temperatures (*P*>0.05) and HDA areas (*P*>0.05) did not differ from resting conditions.

**Fig. 5.**
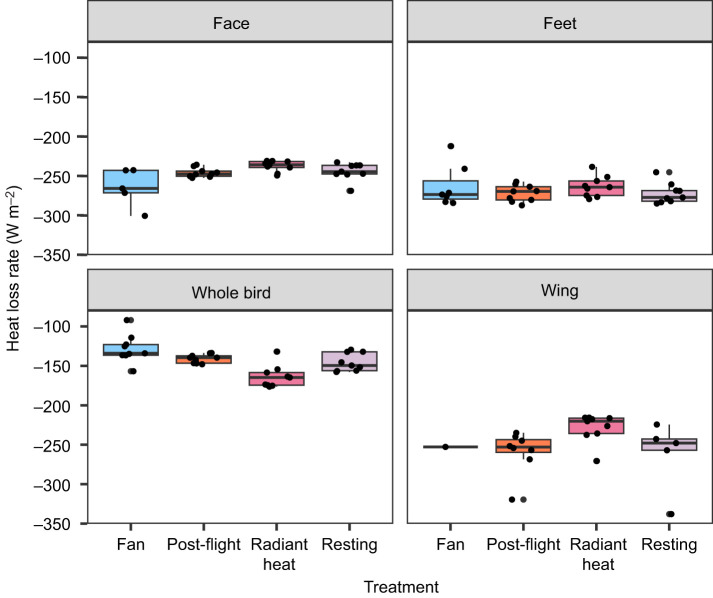
**Calculated rate of heat loss (W m^−2^) in nine doves for each HDA (eye, feet and wing) and ‘whole bird’, an estimate for the entire body as an average across the entire lateral view of the animal, minus the tail.** Graph is colour coded by treatment.

Area-specific heat dissipation estimates indicate that for a given surface area within the active HDA region, the rate of heat loss (W m^−2^) changes significantly (repeated-measures ANCOVA: *P*<0.01; Fig. 5). Area-specific heat dissipation was not significantly different among all treatments in either the feet or the wing (*P*>0.05). At the eye, area-specific heat loss was significantly different between the heatlamp and the fan treatments (*P*=0.012), but not any other treatments.

### The predictive utility of external IR data for core temperatures is moderate

**Fig. 6.**
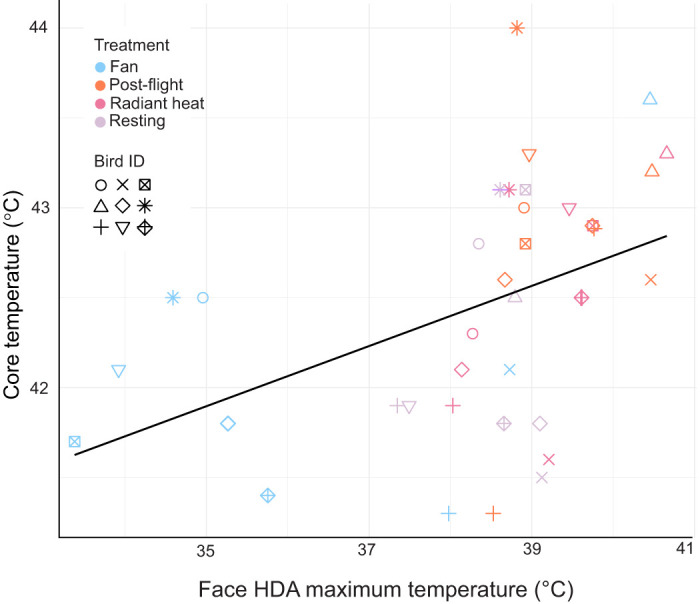
**The relationship between the maximum temperature recorded at the face HDA plotted against core body temperature.** Points are colour coded by individual bird (*n*=9); shape represents the thermal treatment. The linear relationship is plotted as a solid black line; see Results for statistical results.

To explore the predictive power of IR values for core temperature, we compared maximum temperature for the eye HDA with the core body temperatures using a linear mixed effects model (Fig. 6). The first AICc model (Table 1), with 59.4% weighting, suggests that maximum eye temperature is correlated with core body temperatures, but the treatment and interaction between treatment and HDA temperature are both negligible. The second highest weighted model (40.6% weight) found treatment to have an effect, although this model comes at the cost of added complexity. The small difference between the two AIC scores suggests the improved fit of the more complex model likely justifies the additional complexity.

**Table 1.** Results of a corrected Akaike information criteria (AICc) test of models with a weighting over 2% (‘weight’)

Model	Intercept	Eye HDA maximum temperature	Treatment	Interactive HDA:treatment	d.f.	log Likelihood	AICc	Delta	Weight
2	34.99	0.1947	−	−	4	−25.427	60.1	0	0.59
4	34.17	0.2198	+	−	7	−21.452	60.9	0.76	0.404

‘Treatment’ represents the thermal treatments, represented by (+) if it has an effect. The interaction between eye HDA maximum and treatment is null (−) in both models listed. Delta is the difference between AICc values between ranked treatments.

The variation explained by the fixed effects of the above model has a marginal *r*^2^ of 0.36, which includes only the fixed effects of treatment and maximum eye HDA. The conditional *r*^2^ value, including both fixed and random (bird ID) effects, is markedly higher at 0.81.

Predictive power may be strengthened by the addition of more than one HDA. To further explore this potential, we ran a stepwise AIC on a linear model. We found that a combination of the eye, feet and wing mean temperatures were most informative for predicting core temperature (AIC −30.1, RSS 12.49). The model, which did not use individual bird or thermal treatment as factors, resulted in *F*_3,32_=3.68, *r*^2^=0.26.

## DISCUSSION

Our study examined the role of plumage and the modification of temperature and surface area with temperature greater than a 35°C threshold in HDAs in maintaining thermal balance in doves. The animals were tested at multiple thermal regimes, including two externally induced treatments (simulated wind and radiation), and one exercise triggered temperature change. Our results illustrate that the interactive effects of the environment and thermoregulation behaviour influence body temperature in doves.

### Core versus skin-level temperatures

Our results showed that at the skin level, temperatures do not necessarily reflect patterns in the core body temperature (Fig. 2). Post-flight, core temperatures rose by 0.6°C from resting values, but there was no corresponding change in the skin-level temperatures between resting and post-flight (*P*>0.05). Internal heat production owing to locomotion appeared instead to be successfully managed by the active HDAs (see subsequent section), not the skin surface that is covered in plumage, as indicated by the lack of change in overall skin-level temperatures. Evolutionary studies of bill size support the role of HDAs in managing core temperatures (Greenberg et al., 2012). Doves are well adapted to both power-demanding flight styles and hot environments (McKechnie et al., 2016; Schleucher and Withers, 2002), and it is probable that flight trials in this set of experiments did not significantly stress the thermoregulatory capacity of the collared doves. At the degree of flight tested here, doves appear capable of offsetting internal heat production with modifications to active HDA surface area and temperatures.

No observable change from resting temperature values at the skin level during the heat lamp treatment suggested that in the brief periods of heat loading simulated here, the feathers were successful at deflecting radiative heat. Feathers have a demonstrated capacity to deflect IR light, and the radiative heat associated with it (Medina et al., 2018). Dawson and Maloney (2004) showed that feathers out-performed fur at deflecting solar radiation. Thus, our result of not observing a significant change in the skin-level temperature under the radiant heat is not surprising.

In contrast, under the fan treatment there was no significant change in core temperatures from resting values (*P*>0.05), but a marked decrease of 1.6°C at the skin level between resting and fan conditions (*P*<0.01). This decrease indicated that the insulative properties of plumage were compromised by windy conditions. An increase in wind would increase heat loss via forced convection, by the same mechanism as higher flight speeds (Powers et al., 2015). The less effective heat retention may explain the observed relationship of increasing basal metabolic rate with an increase in wind speed (Goldstein, 1983). A comparative study across 156 bird species found evidence for higher nodus density in down feathers, which may increase feather–feather friction to better maintain the insulative layer (Pap et al., 2020). Functional studies of this morphology have not yet been done.

Prior research indicates that thermal conditions have interacting effects. Dark plumaged birds are impacted by solar radiation with higher heat load in comparison with light plumaged birds. This effect is magnified with increased wind velocity: as lighter plumages are more penetrable by radiative heat, they are measurably less impacted by convective cooling (Walsberg et al., 1978). Flight speed and wind speed are inherently linked, which hummingbirds behaviourally capitalize on to thermoregulate effectively (Powers et al., 2015). In natural environments, birds could experience all three of the temperature treatments in this study simultaneously.

### Gradient from core to external

Our study revealed the gradient of decreasing temperatures from core, to skin level under the plumage, to above the plumage, and is novel in measuring these three temperatures simultaneously. Prior research by others visually suggests that birds experiencing artificial wind conditions had lower skin-level temperatures, supporting observations of wind compromising the ability of birds to contain heat, perhaps losing heat directly from the skin via forced convection (Lustick, 1983). Torre-Bueno (1976) and Hirth et al. (1987) measured core and skin-level temperatures with telemetry during flight in a wind tunnel in a starling (*Sturnus vulgaris*) and rock pigeon (*Columba livia*), respectively. In both species, skin temperature appears to track general patterns of core temperature – rising during flight, and lowering post-flight. Our study revealed that these patterns may not accurately track core temperature depending on environmental conditions. It is important to note that our skin-level measurement represented a single PIT tag location, and that there are likely regional differences in the plumage function, and heat dissipation at the skin, throughout the body.

We provide new quantitative details of that gradient, measuring the temperature external to the feathers as well. These data provide direct insight into the thermal flux between core, skin and plumage, and demonstrate the effective insulating capacity of feathers.

### Heat dissipation areas

Our results indicated that the doves modified the surface area and temperature of HDAs to maintain consistent core body temperature. Our observations align with previously recorded changes in blood flow patterns, including increase in bill temperature in toucans when hot (Tattersall et al., 2009), and the well-known ability of birds to modify leg temperature (McQueen et al., 2023; Ryeland et al., 2019; Steen and Steen, 1965), as well as more recent work (Lewden et al., 2023) demonstrating changes in HDAs during flight. Our study further supports that HDA regions change in temperature, with active HDAs exhibiting a corresponding increase in surface areas with higher temperatures. The specific mechanisms birds use to shunt blood differently to their HDAs in an attempt to control body temperature doubtlessly involves modulation of blood flow, but are not well studied and represent a worthwhile area of future research. Further, we visually observed that doves changed the surface area of active HDAs, suggesting that ptiloerection and wing posture may be modulated to allow for useful adjustments in surface area of HDAs.

Following flight trials, we did not observe a change in active HDA surface area (*P*>0.05) or temperature (*P*>0.05), but we did find an increase in the core temperature. This suggests that perhaps our flight performance challenge was not enough to cause significant hyperthermia, or that the birds were able to successfully manage hyperthermia in flight. Recent work on lovebirds indicates that heat dissipation relies heavily on convection in flight (Lewden et al., 2023). Thus, perhaps it is unsurprising that changes in HDAs did not occur post-flight. However, a field study of puffins showed that bill temperature decreases with time post-flight, which suggests the bill is used to help manage and offload heat stored during flight (Schraft et al., 2019).

We found evidence that each active HDA has a distinct maximum temperature (Fig. 4). Although regional heterothermy is no surprise, it is interesting to consider this apparent upper boundary. Notably, dove head temperatures averaged 2°C cooler than the feet (Fig. 4). We hypothesize that this is linked to avoiding overheating the brain, of which the mechanisms are well explored across vertebrates (Caputa, 2004; St-Laurent and Larochelle, 1994). Our result in doves is consistent with patterns observed in hummingbirds (Burgoon et al., 1987), quail (Itsaki-Glucklich and Arad, 1992) and ostrich (Fuller et al., 2003). It is important to note that actual maximum (or minimum) limits may differ than what was observed within our laboratory setting, where ambient temperature was 23^o^C; this observed boundary may vary depending on the environmental conditions.

Our study did not explore behavioural mechanisms of heat dissipation beyond active HDAs (with the notable exception of wing drooping behaviour, which increased surface area of the ‘wing’ HDA observed). Behaviours can include seeking more favourable microsites including shaded areas, minimizing locomotion and panting to promote evaporative water loss. Physical adjustments to the exposure of the HDAs also plays a key role, especially as external conditions become more extreme. For example, birds electively cover their legs (Steen and Steen, 1965), tuck their beaks under plumage (Tattersall et al., 2017), and can adjust feather cover via preening. Birds also directly adjust their plumage via ptiloerection, either allowing for an increase in the insulative layer volume (‘puffing’), or creating active openings in the insulative layer of feathers. All of these behaviours dramatically shift the insulation capabilities of the feathers and HDA zones, and merit further study to improve the understanding of thermoregulatory capacity in birds. Studies examining the role of HDAs and the insulative layer within broader environmental and behavioural contexts (e.g. Pattinson et al., 2020) should be prioritized.

### Do infrared thermography data accurately represent the internal thermal state of the animal?

Thermal imaging has been used as a technique to non-invasively measure surface temperature in wild and captive populations of birds (see McCafferty, 2013 and references therein). Such techniques have been used to directly measure thermoregulation (McCafferty et al., 1998; Powers et al., 2017) as a proxy for stress (Herborn et al., 2015), reproductive demands (Hill et al., 2014), energy expenditure (Tattersall et al., 2020) and even food consumption (Winder et al., 2020). Several recent studies have used the eye region as representative of either core body temperatures (Bakken et al., 2005; Gauchet et al., 2022) or physiological state (Di Giovanni et al., 2022; Herborn et al., 2015; Robertson et al., 2020). Experiments in broiler hens found support for IR data prediction of cloacal temperatures (used as a ‘core body’ temperature proxy) (Cândido et al., 2020), and a field study of roosting red-footed boobies found a strong linear relationship between facial IR data and internal body temperatures measured via implanted temperature loggers (Gauchet et al., 2022). However, the present study was unable to strongly identify a relationship between external measurements of HDA temperature and the internal core temperature, requiring all possible data input for an *r*^2^ of 0.26. We found limited support for a robust relationship between the surface temperatures of a single HDA (the ‘face’) maximum temperature and core body temperature. This relationship improved when either individual ID or thermal treatment was accounted for statistically. In field conditions, both individual ID and microclimate conditions can be difficult or impossible to determine. As such, we raise concerns with using a single HDA as a proxy for core body temperatures.

Coupling our statistical analyses with the results of Tabh et al. (2021), who found that IR measurement values were susceptible to orientation, and Soravia et al. (2022), who did not find support for measurement repeatability in orbital temperatures in wild birds, leads us to promote a cautious approach when extrapolating field values to represent internal core condition of the animals being measured. However, there are obvious relationships between external thermal patterns and the physiological and behavioural experience of the animals that are valuably interpreted using surface-level measurements (McCafferty, 2013).

### Conclusion

Core body temperatures remained consistent across heating and cooling treatments but increased following flight. Core temperature was not reflected by skin-level temperatures, suggesting that overheating was effectively managed by modifying HDAs. We found evidence that doves alter both the overall temperature and surface area of the three major active HDAs (face, wing and feet), to maintain consistent core body temperatures under induced temperature challenges.

## Supplementary Material

10.1242/jeb.248200_sup1 Supplementary information

Dataset 1. This data file consists of two sheets and contains all data used in the manuscript.

## References

[JEB248200C67] Alexander, R. M. (2002). The merits and implications of travel by swimming, flight and running for animals of different sizes. *Int. Comp. Biol.* 42, 1060-1064. doi:10.1093/icb/42.5.1060 21680388

[JEB248200C1] Arad, Z., Midtgård, U. and Bernstein, M. H. (1989). Thermoregulation in turkey vultures: vascular anatomy, arteriovenous heat exchange, and behavior. *Condor* 91, 505-514. 10.2307/1368103

[JEB248200C2] Bakken, G. S., Van Sant, M. J., Lynott, A. J. and Banta, M. R. (2005). Predicting small endotherm body temperatures from scalp temperatures. *J. Therm. Biol.* 30, 221-228. 10.1016/j.jtherbio.2004.11.005

[JEB248200C3] Bates, D., Mächler, M., Bolker, B. and Walker, S. (2015). Fitting linear mixed-effects models using lme4. *J. Stat. Softw.* 67, 1-48. 10.18637/jss.v067.i01

[JEB248200C4] Bourne, A. R. and Soravia, C. (2023). Huddling in the heat? Rarely seen thermoregulatory behaviours as southern pied babblers *Turdoides bicolor* compete for cool microsites. *Ostrich* 94, 217-222. 10.2989/00306525.2023.2269477

[JEB248200C5] Bourne, A. R., Ridley, A. R. and Cunningham, S. J. (2023). Helpers don't help when it's hot in a cooperatively breeding bird, the southern pied babbler. *Behav. Ecol.* 34, 562-570. 10.1093/beheco/arad023 37434640 PMC10332451

[JEB248200C6] Burgoon, D. A., Kilgore, D. L. and Motta, P. J. (1987). Brain temperature in the calliope hummingbird (*Stellula calliope*): a species lacking a rete mirabile ophthalmicum. *J. Comp. Physiol. B* 157, 583-588. 10.1007/BF00700978

[JEB248200C7] Butler, P. J. (1991). Exercise in birds. *J. Exp. Biol.* 160, 233-262. 10.1242/jeb.160.1.233

[JEB248200C8] Cândido, M. G. L., Tinôco, I. F. F., Albino, L. F. T., Freitas, L. C. S. R., Santos, T. C., Cecon, P. R. and Gates, R. S. (2020). Effects of heat stress on pullet cloacal and body temperature. *Poult. Sci.* 99, 2469-2477. 10.1016/j.psj.2019.11.062 32359582 PMC7597385

[JEB248200C9] Caputa, M. (2004). Selective brain cooling: a multiple regulatory mechanism. *J. Therm. Biol.* 29, 691-702. 10.1016/j.jtherbio.2004.08.079

[JEB248200C10] Dawson, T. J. and Maloney, S. K. (2004). Fur versus feathers: the different roles of red kangaroo fur and emu feathers in thermoregulation in the Australian arid zone. *Aust. Mammal.* 26, 145-151. 10.1071/AM04145

[JEB248200C11] Di Giovanni, J., Fawcett, T. W., Templeton, C. N., Raghav, S. and Boogert, N. J. (2022). Urban gulls show similar thermographic and behavioral responses to human shouting and conspecific alarm calls. *Front. Ecol. Evol.* 10, 891985. 10.3389/fevo.2022.891985

[JEB248200C12] Dove, C. J. and Agreda, A. (2007). Differences in plumulaceous feather characters of dabbling and diving ducks. *Condor* 109, 192-199. 10.1093/condor/109.1.192

[JEB248200C13] du Plessis, K. L., Martin, R. O., Hockey, P. A. R., Cunningham, S. J. and Ridley, A. R. (2012). The costs of keeping cool in a warming world: implications of high temperatures for foraging, thermoregulation and body condition of an arid-zone bird. *Glob. Change Biol.* 18, 3063-3070. 10.1111/j.1365-2486.2012.02778.x 28741828

[JEB248200C14] Edgar, J. L., Lowe, J. C., Paul, E. S. and Nicol, C. J. (2011). Avian maternal response to chick distress. *Proc. R. Soc. B* 278, 3129-3134. 10.1098/rspb.2010.2701 PMC3158930 21389025

[JEB248200C15] Fuller, A., Kamerman, P. R., Maloney, S. K., Mitchell, G. and Mitchell, D. (2003). Variability in brain and arterial blood temperatures in free-ranging ostriches in their natural habitat. *J. Exp. Biol.* 206, 1171-1181. 10.1242/jeb.00230 12604577

[JEB248200C16] Gauchet, L., Jaeger, A. and Grémillet, D. (2022). Using facial infrared thermography to infer avian body temperatures in the wild. *Mar. Biol.* 169, 57. 10.1007/s00227-022-04041-y

[JEB248200C17] Gerson, A. R., Smith, E. K., Smit, B., McKechnie, A. E. and Wolf, B. O. (2014). The impact of humidity on evaporative cooling in small desert birds exposed to high air temperatures. *Physiol. Biochem. Zool.* 87, 782-795. 10.1086/678956 25461643

[JEB248200C18] Goldstein, D. L. (1983). Effect of wind on avian metabolic rate with particular reference to Gambel's quail. *Physiol. Zool.* 56, 485-492. 10.1086/physzool.56.4.30155871

[JEB248200C19] Greenberg, R., Cadena, V., Danner, R. M. and Tattersall, G. (2012). Heat loss may explain bill size differences between birds occupying different habitats. *PLoS ONE* 7, e40933. 10.1371/journal.pone.0040933 22848413 PMC3405045

[JEB248200C20] Herborn, K. A., Graves, J. L., Jerem, P., Evans, N. P., Nager, R., McCafferty, D. J. and McKeegan, D. E. (2015). Skin temperature reveals the intensity of acute stress. *Physiol. Behav.* 152, 225-230. 10.1016/j.physbeh.2015.09.032 26434785 PMC4664114

[JEB248200C21] Hill, D. L., Lindström, J., McCafferty, D. J. and Nager, R. G. (2014). Female but not male zebra finches adjust heat output in response to increased incubation demand. *J. Exp. Biol.* 217, 1326-1332. 10.1242/jeb.095323 24363422

[JEB248200C22] Hirth, K.-D., Biesel, W. and Nachtigall, W. (1987). Pigeon flight in a wind tunnel: III. Regulation of body temperature. *J. Comp. Physiol. B* 157, 111-116. 10.1007/BF00702735

[JEB248200C23] Itsaki-Glucklich, S. and Arad, Z. (1992). The effect of dehydration on brain temperature regulation in Japanese quail (*Coturnix coturnix japonica*). *Comp. Biochem. Physiol. Comp. Physiol.* 101, 583-588. 10.1016/0300-9629(92)90512-O 1348680

[JEB248200C24] Lewden, A., Bishop, C. M. and Askew, G. N. (2023). How birds dissipate heat before, during and after flight. *J. R. Soc. Interface* 20, 20230442. 10.1098/rsif.2023.0442 38086401 PMC10715914

[JEB248200C25] Lucas, A. and Stettenheim, P. (1972). *Avian Anatomy–Integument. Agricultural Handbook 362*, pp. 485-635. Washington, DC: US Department of Agriculture.

[JEB248200C26] Lustick, S. I. (1983). Cost–benefit of thermoregulation in birds: influences of posture, microhabitat selection, and color. In *Behavioral Energetics: The Cost of Survival in Vertebrates* (ed. W. Aspey and S. I. Lustick), pp. 265-294. Columbus, OH: Ohio State University Press.

[JEB248200C27] Martineau, L. and Larochelle, J. (1988). The cooling power of pigeon legs. *J. Exp. Biol.* 136, 193-208. 10.1242/jeb.136.1.193

[JEB248200C28] McCafferty, D. J. (2013). Applications of thermal imaging in avian science. *Ibis* 155, 4-15. 10.1111/ibi.12010

[JEB248200C29] McCafferty, D. J., Moncrieff, J. B., Taylor, I. R. and Boddie, G. F. (1998). The use of IR thermography to measure the radiative temperature and heat loss of a barn owl (Tyto alba). *J. Therm. Biol.* 23, 311-318. 10.1016/S0306-4565(98)00022-9

[JEB248200C30] McCafferty, D. J., Gilbert, C., Paterson, W., Pomeroy, P. P., Thompson, D., Currie, J. I. and Ancel, A. (2011). Estimating metabolic heat loss in birds and mammals by combining infrared thermography with biophysical modelling. *Comp. Biochem. Physiol. A Mol. Integr. Physiol.* 158, 337-345. 10.1016/j.cbpa.2010.09.012 20869456

[JEB248200C31] McFarland, D. and Baher, E. (1968). Factors affecting feather posture in the barbary dove. *Anim. Behav.* 16, 171-177. 10.1016/0003-3472(68)90127-9 5689100

[JEB248200C32] McFarland, D. and Budgell, P. (1970). The thermoregulatory role of feather movements in the Barbary dove (*Streptopelia risoria*). *Physiol. Behav.* 5, 763-771. 10.1016/0031-9384(70)90276-3 5522492

[JEB248200C33] McKechnie, A. E., Whitfield, M. C., Smit, B., Gerson, A. R., Smith, E. K., Talbot, W. A., McWhorter, T. J. and Wolf, B. O. (2016). Avian thermoregulation in the heat: efficient evaporative cooling allows for extreme heat tolerance in four Southern Hemisphere columbids. *J. Exp. Biol.* 219, 2145-2155. 10.1242/jeb.146563 27207640

[JEB248200C34] McQueen, A., Barnaby, R., Symonds, M. R. E. and Tattersall, G. J. (2023). Birds are better at regulating heat loss through their legs than their bills: implications for body shape evolution in response to climate. *Biol. Lett.* 19, 20230373. 10.1098/rsbl.2023.0373 37990562 PMC10663788

[JEB248200C35] Medina, I., Newton, E., Kearney, M. R., Mulder, R. A., Porter, W. P. and Stuart-Fox, D. (2018). Reflection of near-infrared light confers thermal protection in birds. *Nat. Commun.* 9, 3610. 10.1038/s41467-018-05898-8 30190466 PMC6127310

[JEB248200C36] Nakagawa, S. and Schielzeth, H. (2013). A general and simple method for obtaining *R*^2^ from generalized linear mixed-effects models. *Methods Ecol. Evol.* 4, 133-142. 10.1111/j.2041-210x.2012.00261.x

[JEB248200C37] Nord, A. and Nilsson, J. (2019). Heat dissipation rate constrains reproductive investment in a wild bird. *Funct. Ecol.* 33, 250-259. 10.1111/1365-2435.13243

[JEB248200C38] Pap, P. L., Osváth, G., Daubner, T., Nord, A. and Vincze, O. (2020). Down feather morphology reflects adaptation to habitat and thermal conditions across the avian phylogeny. *Evolution* 74, 2365-2376. 10.1111/evo.14075 32748406

[JEB248200C39] Pattinson, N. B., Thompson, M. L., Griego, M., Russell, G., Mitchell, N. J., Martin, R. O., Wolf, B. O., Smit, B., Cunningham, S. J., McKechnie, A. E. et al. (2020). Heat dissipation behaviour of birds in seasonally hot arid-zones: are there global patterns? *J. Avian Biol.* 51, e02350. 10.1111/jav.02350

[JEB248200C40] Powers, D. R., Tobalske, B. W., Wilson, J. K., Woods, H. A. and Corder, K. R. (2015). Heat dissipation during hovering and forward flight in hummingbirds. *R. Soc. Open Sci.* 2, 150598. 10.1098/rsos.150598 27019742 PMC4807464

[JEB248200C41] Powers, D. R., Langland, K. M., Wethington, S. M., Powers, S. D., Graham, C. H. and Tobalske, B. W. (2017). Hovering in the heat: effects of environmental temperature on heat regulation in foraging hummingbirds. *R. Soc. Open Sci.* 4, 171056. 10.1098/rsos.171056 29308244 PMC5750011

[JEB248200C42] Robertson, J. K., Mastromonaco, G. and Burness, G. (2020). Evidence that stress-induced changes in surface temperature serve a thermoregulatory function. *J. Exp. Biol.* 223, jeb213421. 10.1242/jeb.213421 31974220

[JEB248200C43] Ryeland, J., Weston, M. A. and Symonds, M. R. E. (2019). Leg length and temperature determine the use of unipedal roosting in birds. *J. Avian Biol.* 50, e02008. 10.1111/jav.02008

[JEB248200C44] Schleucher, E. and Withers, P. C. (2002). Metabolic and thermal physiology of pigeons and doves. *Physiol. Biochem. Zool.* 75, 439-450. 10.1086/342803 12529845

[JEB248200C45] Schraft, H. A., Whelan, S. and Elliott, K. H. (2019). Huffin’ and puffin: seabirds use large bills to dissipate heat from energetically demanding flight. *J. Exp. Biol.* 222, jeb212563. 10.1242/jeb.212563 31624096

[JEB248200C46] Silva, J. P., Catry, I., Palmeirim, J. M. and Moreira, F. (2015). Freezing heat: thermally imposed constraints on the daily activity patterns of a free-ranging grassland bird. *Ecosphere* 6, 1-13. 10.1890/ES14-00454.1

[JEB248200C47] Soravia, C., Ashton, B. J. and Ridley, A. R. (2022). Periorbital temperature responses to natural air temperature variation in wild birds. *J. Therm. Biol.* 109, 103323. 10.1016/j.jtherbio.2022.103323 36195398

[JEB248200C66] Speakman, J. R. and Król, E. (2005). Maximal heat dissipation capacity and hyperthermia risk: neglected key factors in the ecology of endotherms. *J. Anim. Ecol. B.* 79, 726-746. 10.1111/j.1365-2656.2010.01689.x 20443992

[JEB248200C48] St-Laurent, R. and Larochelle, J. (1994). The cooling power of the pigeon head. *J. Exp. Biol.* 194, 329-339. 10.1242/jeb.194.1.329 9317904

[JEB248200C49] Steen, I. and Steen, J. B. (1965). The importance of the legs in the thermoregulation of birds. *Acta Physiol. Scand.* 63, 285-291. 10.1111/j.1748-1716.1965.tb04067.x 14324065

[JEB248200C50] Tabh, J. K., Burness, G., Wearing, O. H., Tattersall, G. J. and Mastromonaco, G. F. (2021). Infrared thermography as a technique to measure physiological stress in birds: body region and image angle matter. *Physiol. Rep.* 9, e14865. 10.14814/phy2.14865 34057300 PMC8165734

[JEB248200C51] Tapper, S., Nocera, J. J. and Burness, G. (2020). Heat dissipation capacity influences reproductive performance in an aerial insectivore. *J. Exp. Biol.* 223, jeb222232. 10.1242/jeb.222232 32321750

[JEB248200C52] Tattersall, G. J., Andrade, D. V. and Abe, A. S. (2009). Heat exchange from the toucan bill reveals a controllable vascular thermal radiator. *Science* 325, 468-470. 10.1126/science.1175553 19628866

[JEB248200C53] Tattersall, G. J., Arnaout, B. and Symonds, M. R. E. (2017). The evolution of the avian bill as a thermoregulatory organ. *Biol. Rev.* 92, 1630-1656. 10.1111/brv.12299 27714923

[JEB248200C54] Tattersall, G. J., Chaves, J. A. and Danner, R. M. (2018). Thermoregulatory windows in Darwin's finches. *Funct. Ecol.* 32, 358-368. 10.1111/1365-2435.12990

[JEB248200C55] Tattersall, G. J., Danner, R. M., Chaves, J. A. and Levesque, D. L. (2020). Activity analysis of thermal imaging videos using a difference imaging approach. *J. Therm. Biol.* 91, 102611. 10.1016/j.jtherbio.2020.102611 32716861

[JEB248200C56] Terrill, R. S. and Shultz, A. J. (2023). Feather function and the evolution of birds. *Biol. Rev.* 98, 540-566. 10.1111/brv.12918 36424880

[JEB248200C57] Torre-Bueno, J. R. (1976). Temperature regulation and heat dissipation during flight in birds. *J. Exp. Biol.* 65, 471-482. 10.1242/jeb.65.2.471 1003090

[JEB248200C58] Venables, W. and Ripley, B. (2002). *Modern Applied Statistics with S*. New York: Springer.

[JEB248200C59] Walsberg, G. E. (1983). Coat color and solar heat gain in animals. *Bioscience* 33, 88-91. 10.2307/1309169

[JEB248200C60] Walsberg, G. E., Campbell, G. S. and King, J. R. (1978). Animal coat color and radiative heat gain: a re-evaluation. *J. Comp. Physiol.* 126, 211-222. 10.1007/BF00688930

[JEB248200C61] Ward, S., Rayner, J. M. V., Möller, U., Jackson, D. M., Nachtigall, W. and Speakman, J. R. (1999). Heat transfer from starlings *Sturnus vulgaris* during flight. *J. Exp. Biol.* 202, 1589-1602. 10.1242/jeb.202.12.1589 10333506

[JEB248200C62] Welch, K. C. Jr, Altshuler, D. L. and Suarez, R. K. (2007). Oxygen consumption rates in hovering hummingbirds reflect substrate-dependent differences in P/O ratios: carbohydrate as a ‘premium fuel’. *J. Exp. Biol.* 210, 2146-2153. 10.1242/jeb.005389 17562888

[JEB248200C63] Wells, D. J. (1993). Muscle performance in hovering hummingbirds. *J. Exp. Biol.* 178, 39-57. 10.1242/jeb.178.1.39

[JEB248200C64] Winder, L. A., White, S. A., Nord, A., Helm, B. and McCafferty, D. J. (2020). Body surface temperature responses to food restriction in wild and captive great tits. *J. Exp. Biol.* 223, jeb220046. 10.1242/jeb.220046 32312718

[JEB248200C65] Wolf, B. O. and Walsberg, G. E. (2000). The role of the plumage in heat transfer processes of birds. *Am. Zool.* 40, 575-584. 10.1093/icb/40.4.575

