# Supplementary material for: The role of plumage and heat dissipation areas in thermoregulation in doves
Source: J Exp Biol. 2025 Feb 20;228(4):JEB248200. doi: 10.1242/jeb.248200 (PMC12000677; doi:10.1242/jeb.248200)
Supplement: Supplementary information [file jexbio-228-248200-s1.pdf]

Dataset 1. This data file consists of two sheets and contains all data used in the manuscript.

FLIR Data (Sheet 1)
This sheet contains data collected from FLIR images.
Explanation of columns:

- "Date of collection"
  date the data was collected in DD-MM format

- "bird ID"
  ID of the collared dove used based on their coloured ring band patterns. The following nine birds were used in this collection: BlackBlack, Orange, Yellow, Red, WhiteWhite, RedBlack, Blue, BlueWhite, and BlueBlue

- "treatment"
  thermal treatment the animal was subjected to. Briefly: "resting" is the bird with no thermal manipulations, "flight" is following a period of 10 perch-to-perch flights, "heatlamp" is following ten minutes under a heating element, and "fan" is following ten minutes of cooling via a fan. See manuscript text for further details regarding each treatment.

- "type of measurement"
  indicates whether the values were collected from a targeted 'hda' (heat dissipation area) or are an average taken across the entire body of the bird ('wholebird')

- "hda"
  'heat dissipation area' measured from the FLIR images. 'Eye' is the area of the face, including the beak, 'wing' is the area surrounding the wing and wing-body joint, 'feet' is the unfeathered feet, and 'wholebird' is a measure of the entire body of the bird, without the tail. See text for detailed descriptions.

- "area (cms)"
  the measured area of the heat dissipation area (HDA) over the designated threshold (generally 35 C, see text) measured from the FLIR images. Unites are cm.

- "mean (*C)"
  mean temperature, in degrees Celsius, of the heat dissipation area (HDA)

- "max (*C)
  maximum temperature, in degrees Celsius, of the heat dissipation area (HDA)

PIT Data (Sheet 2)
This sheet contains data collected from thermal PIT tags in and on the bird.

Explanation of columns:

- "Date of collection"
  date the data was collected in DD-MM format

- "bird ID"
  ID of the collared dove used based on their coloured ring band patterns. The following nine birds were used in this collection: BlackBlack, Orange, Yellow, Red, WhiteWhite, RedBlack, Blue, BlueWhite, and BlueBlue

- "treatment"
  thermal treatment the animal was subjected to. Briefly: "resting" is the bird with no thermal manipulations, "flight" is following a period of 10 perch-to-perch flights, "heatlamp" is following ten minutes under a heating element, and "fan" is following ten minutes of cooling via a fan. See manuscript text for further details regarding each treatment.

- "PIT location"
  Two PIT tags were used, and this column indicates which PIT tag the data came from. "Internal" PIT tag was surgically imbedded in to the animal and represents a core body temperature. "external" PIT tag was placed on the skin with glue under the feather layer. See methods section for further details on PIT tag placement.

- "temperature (C)"
  Temperature, in degrees Celsius, collected from the PIT tag. See text for further details on how temperature values were identified.

Available for download at
https://journals.biologists.com/jeb/article-lookup/doi/10.1242/jeb.248200#supplementary-data
